# Effects of *Candidatus* Liberibacter asiaticus infection on metagenome of *Diaphorina citri* gut endosymbiont

**DOI:** 10.1038/s41597-023-02345-2

**Published:** 2023-07-21

**Authors:** Qi Pan, Shi-Jiang Yu, Shuang Lei, Si-Chen Li, Li-Li Ding, Liu Liu, Lu-Yan Cheng, Ren Luo, Cui-Yun Lei, Bing-Hai Lou, Lin Cong, Hao-Qiang Liu, Xue-Feng Wang, Chun Ran

**Affiliations:** 1 Citrus Research Institute, Southwest University/Chinese Academy of Agricultural Sciences, National Engineering Research Center for Citrus, Chongqing, 400712 China; 2 Jianli Agriculture Technology Promotion Center, Jingzhou, Hubei 433300 China; 3 Guangxi Key Laboratory of Citrus Biology, Guangxi Academy of Specialty Crops, Guilin, Guangxi 541004 P.R. China

**Keywords:** Microbiology, Metagenomics

## Abstract

Asian citrus psyllid (*Diaphorina citri*, *D. citri*) is the important vector of “*Candidatus* Liberibacter asiaticus” (*C*Las), associated with Huanglongbing, the most devastating citrus disease worldwide. *C*Las can affect endosymbiont abundance of *D. citri*. Here, we generated the high-quality gut endosymbiont metagenomes of *Diaphorina citri* on the condition of *C*Las infected and uninfected. The dataset comprised 6616.74 M and 6586.04 M raw reads, on overage, from *C*Las uninfected and infected psyllid strains, respectively. Taxonomic analysis revealed that a total of 1046 species were annotated with 10 Archaea, 733 Bacteria, 234 Eukaryota, and 69 Viruses. 80 unique genera in *C*Las infected *D. citri* were identified. DIAMOND software was used for complement function research against various functional databases, including Nr, KEGG, eggNOG, and CAZy, which annotated 84543 protein-coding genes. These datasets provided an avenue for further study of the interaction mechanism between *C*Las and *D. citri*.

## Background & Summary

Huanglongbing (HLB) is the most destructive citrus disease around the world. Asian citrus psyllid (ACP), *Diaphorina citri* Kuwayama, is the important carrier of *Candidatus* Liberibacter asiaticus (*C*Las), the causal agent of HLB. ACP has been found in many countries such as the Arabian Peninsula, Indian Subcontinent, South East and South West Asia, the USA, Central and South America, and the Indian Ocean islands of Mauritius and R´eunion^1,2^. *C*Las can infect many organs and tissues of ACP including the alimentary canal, hemolymph, hemocytes, muscle tissue, fat tissue, bacteriome, neural tissue, epidermis, reproductive organs, and salivary glands^2^, which affects not only the ACP’s physiological function, but also its endosymbiont communities^3,4^. However, it is little known about the relation between the change of endosymbiont communities and the physiological function alteration of ACP after being infected by *C*Las.

**Fig. 1:**
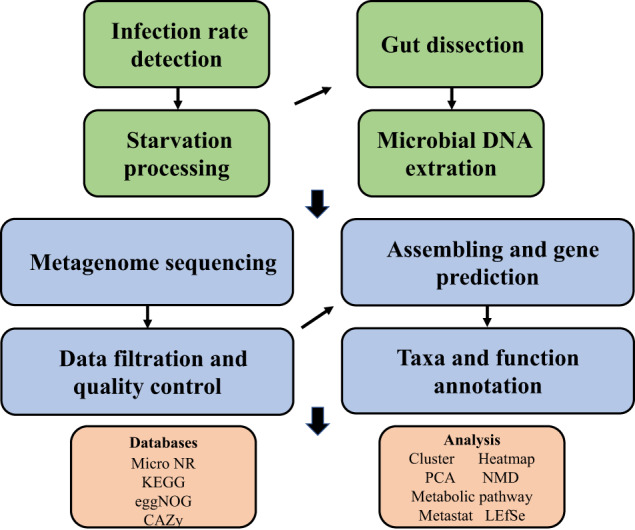
Flow chart used to process psyllid gut samples to get metagenomes.

**Fig. 2:**
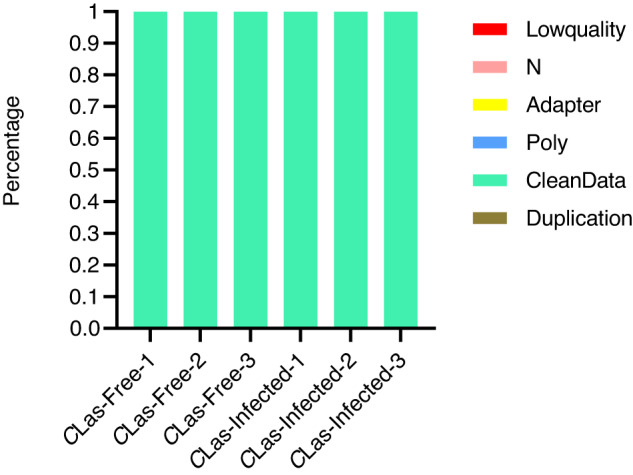
Data preprocessing statistic map. Adapter: reads with adapters; Low Quality: low-quality reads; PolyA: number of reads containing polyA (%); N: single-ended reads containing more than 10% of N bases; Duplication: total length of the removed repeat reads sequence.

Analysis of the change of endosymbiont communities is the basis for understanding the interaction between *C*Las and ACP. Recent studies have shown that three predominant endosymbionts including *Candidatus* Carsonella ruddii, *Candidatus* Profftella, and *Wolbachia* were harbored in ACP^5,6^. These endosymbionts play different roles in participating in the ACP’s physiological activities. For instance, Carsonella has been considered to be a nutritional provider and Profftella has been verified to be a defender because of producing diaphorin^6,7^. *Wolbachia* titer has been demonstrated to have a positive correlation with *C*Las titer^8^. However, the key function of *Wolbachia* in ACP remains unclear. Data of endosymbiont communities in ACP have been reported based on 16 S rRNA sequencing^9,10^. Despite bacteria communities of genus level can be verified by conserved regions analysis in 16 S rRNA hypervariable regions, detailed composition on endosymbiont at species level comprised of bacteria, archaea, and Eukaryota and function characteristics are still needed. High-throughput metagenome sequencing technology can provide new views from a nearly complete composition of endosymbiont communities^11^.

**Fig. 3:**
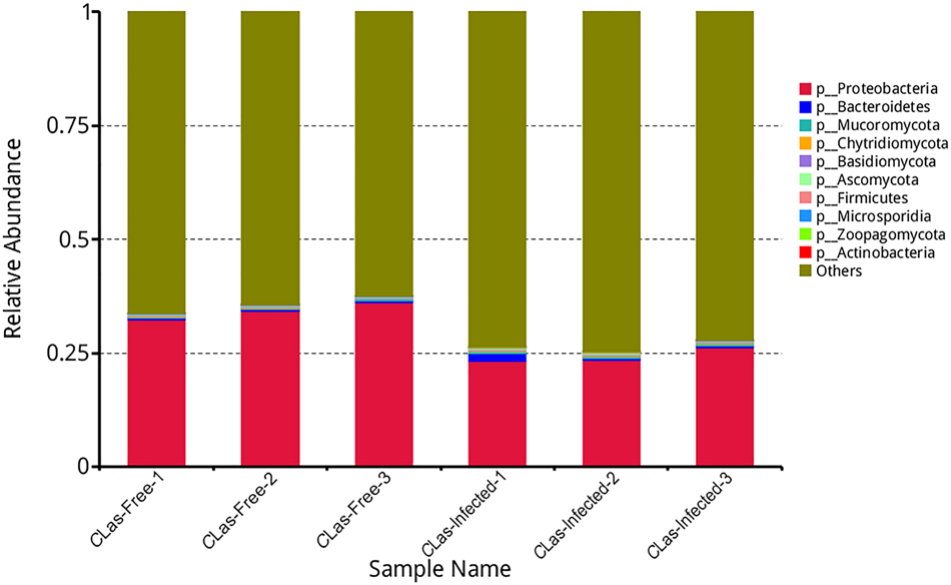
The composition of microbiota at the phylum level. Taxonomic assignments of the 10 most abundant taxa are presented.

**Fig. 4:**
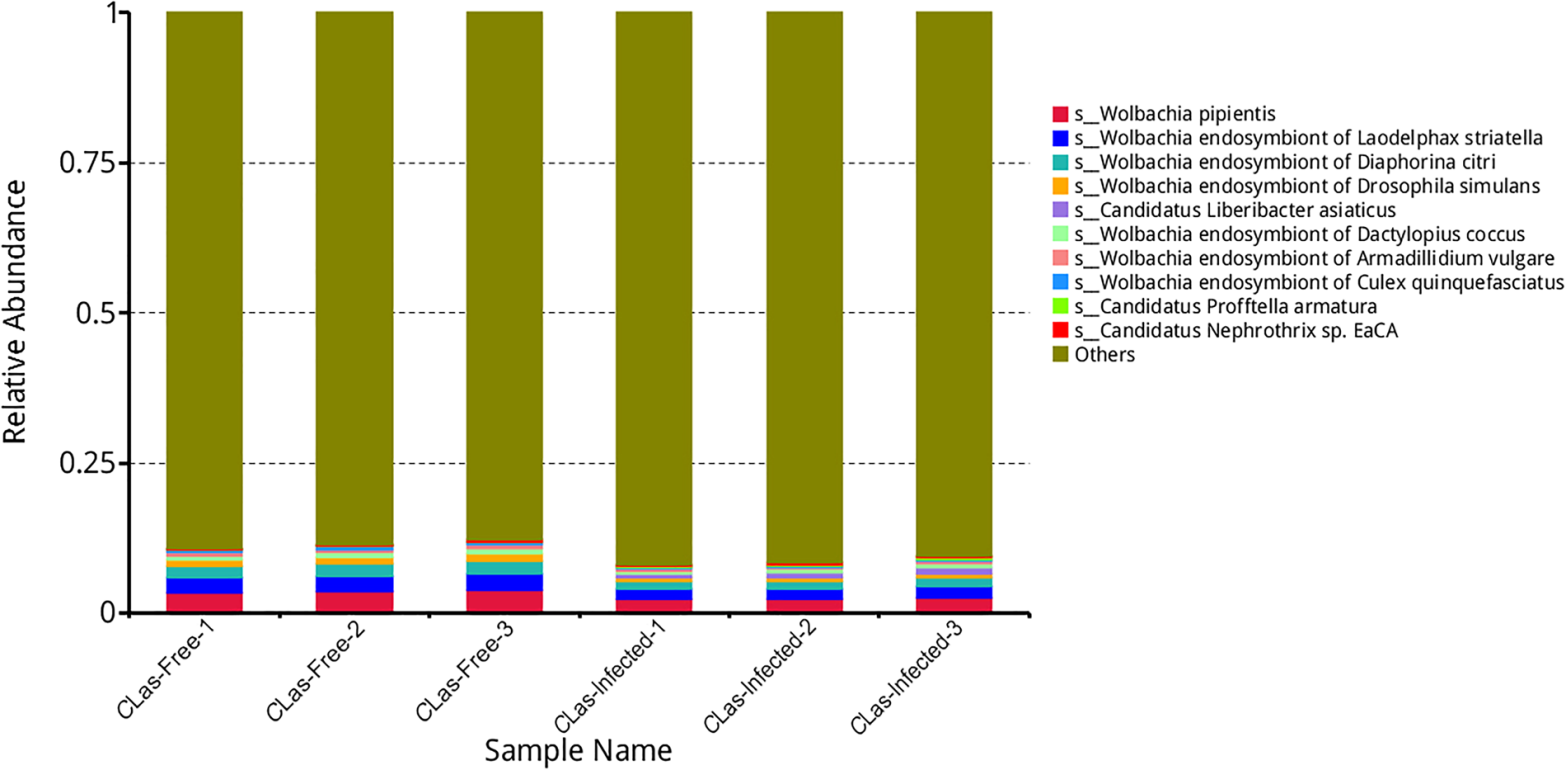
The composition of microbiota at species level. Taxonomic assignments of the 10 most abundant taxa are presented.

**Fig. 5:**
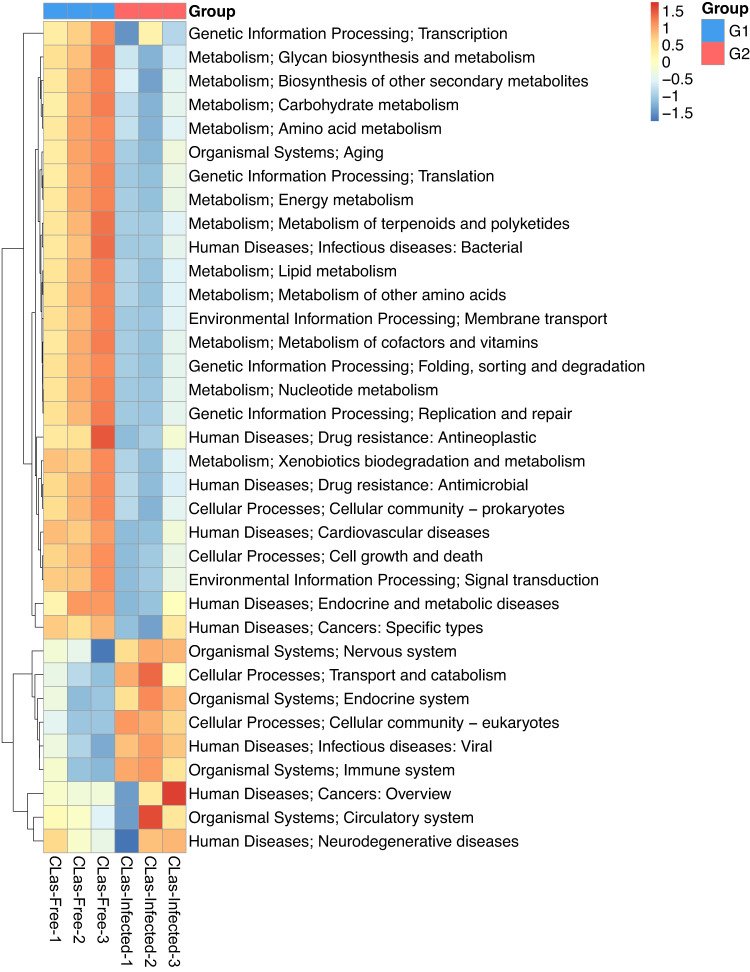
Hierarchically clustered heat map analysis of the highly represented KOs category of KEGG level 2.

**Fig. 6:**
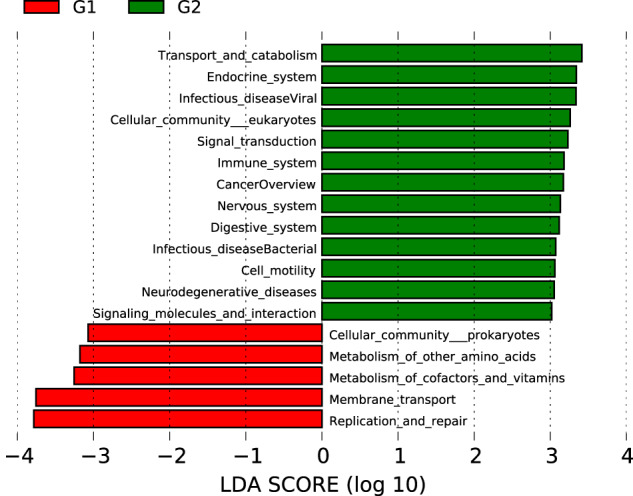
LEfSe analysis of the category of KEGG level 2 that differ between HLB-free and HLB-infected *D. citri*. G1: HLB-free *D. citri*, G2: HLB-infected *D. citri*.

**Fig. 7:**
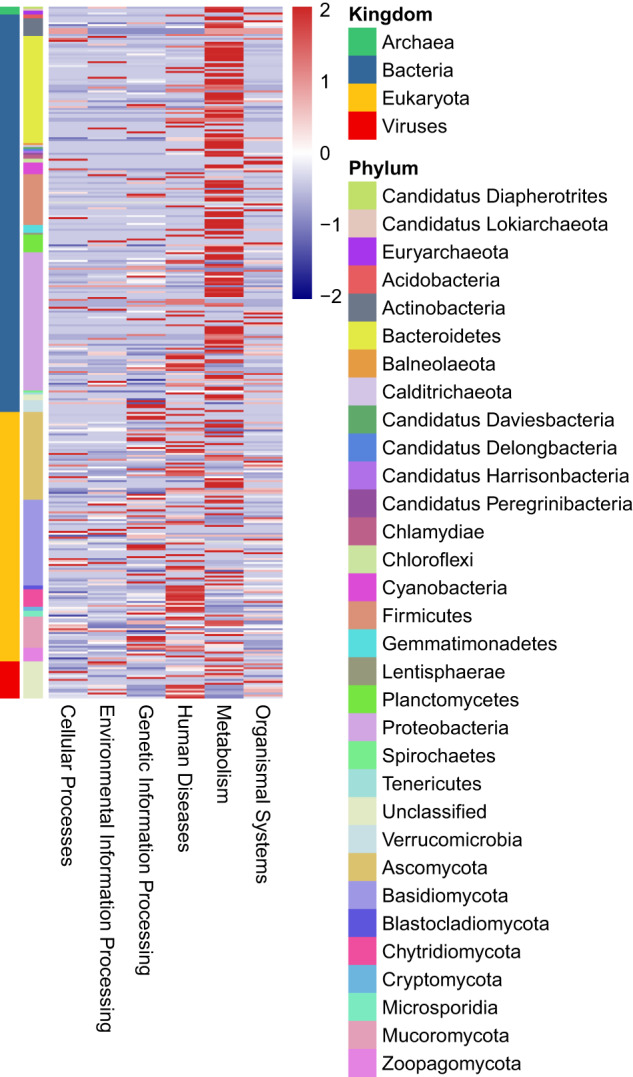
Heatmap of the distribution of KEGG. The horizontal axis represents 6 different kinds of KEGG, and the different colors of the vertical axis represent different endosymbiont taxonomic information.

Here, we characterized two endosymbiont metagenomes from the ACP strains before and after infection by *C*Las, which were named *C*Las-free and *C*Las-infected, respectively. Sample information was presented in Table 1. Raw data among 6175.81–7204.40 M were produced via metagenomic sequencing, in total. After filtration of the contaminated reads, clean data of six samples were between 6168.97 M and 7193.66 M. Comparatively, approximate 17.41–18.85 M data of microbiota from ACP were obtained using 16 s amplicon sequencing, suggesting that our data is more complete than the previous results^10^. Based on the metagenomes assembling, the number of scaffolds was separately distributed into 185390–196132 with 36.81–37.49% GC content in *C*Las-free and *C*Las-infected. The Q20 values and Q30 values were all above 96% and 90% respectively, and the N50 values were all above 1000 bp (Table 2). Table 3 generalized the overview of the composition of gut endosymbiont communities between *C*Las-free and *C*Las-infected and Table 4 summarized the gene number with function annotated after alignment in different functional databases. Figure 1 summarized the process of experiment.The endosymbiont orders with biomarkers were differed between the two groups, where Proteobacteria and *Wolbachia* pipientis were the most abundant in taxonomic phylum and species, respectively (Figs. 3, 4). KEGG orthology groups (KOs) relating to the category of metabolism were the most abundant in the intestinal microbiota of *C*Las-free and *C*Las-infected *D. citri*, such as biosynthesis and metabolism of amino acid, cofactors, vitamins, and glycan (Fig. 5). Five categories including the metabolism of other amino acids, cofactors, and vitamins were enriched in *C*Las-free *D. citri* samples and thirteen categories including immune and nervous systems were enriched in *C*Las-infected *D. citri* samples, indicating that two different gut endosymbionts of *D. citri* had a distinct contribution for host function compensation (Fig. 6). These KOs were unevenly found in the metagenomic of the taxa. For instance, KOs belonging to the category of metabolism were enriched in the bacteria of Firmicutes and Proteobacteria and the Eukaryota of Ascomycotab (Fig. 7).

**Table 1:** Sample information in this study.

Sample	Biome	Feature	Material	Geographical location	GeoPosition	Protocol
*C*Las-Free	Insect gut	HLB-free	Gut tissue	Guilin of Guangxi province, China	110.328611, 25.011667, 5 m	Metagenome
*C*Las-Infected	Insect gut	HLB-infected	Gut tissue	Guilin of Guangxi province, China	110.325470, 25.270744, 5 m	Metagenome

**Table 2:** Statistics of metagenomic sequencing data.

Sample	*C*Las-Free-1	*C*Las-Free-2	*C*Las-Free-3	*C*Las-Infected-1	*C*Las-Infected-2	*C*Las-Infected-3
**RawData (M)**	6847.57	6478.32	6524.33	7204.4	6377.92	6175.81
**CleanData (M)**	6831.02	6469.84	6517.59	7193.66	6370.68	6168.97
**Q20 (%)**	96.97	96.59	96.75	96.88	96.22	96.28
**Q30 (%)**	92.52	91.47	91.81	92.34	90.84	90.98
**GC (%)**	36.96	36.84	36.81	37.49	37.22	37.28
**Total length (bp)**	1.86E + 08	1.88E + 08	1.89E + 08	1.93E + 08	1.86E + 08	190367106
**Number of scaffolds**	187172	185390	185617	196132	187433	187492
**Average length (bp)**	991.18	1011.93	1019.24	985.79	990.54	1015.33
**N50 (bp)**	1022	1054	1067	1009	1016	1057
**N90 (bp)**	568	572	573	564	566	571
**Largest length (bp)**	27372	89417	34284	130775	130777	130774
**Effective (%)**	99.76	99.87	99.9	99.85	99.89	99.89

**Table 3:** Statistics of domain coverage of gene.

Taxonomy	Gene number (%)					
	*C*Las-Free-1	*C*Las-Free-2	*C*Las-Free-3	*C*Las-Infected-1	*C*Las-Infected-2	*C*Las-Infected-3
**Archaea**	28 (0.01%)	28 (0.01%)	28 (0.01%)	28 (0.01%)	28 (0.01%)	28 (0.01%)
**Bacteria**	6106 (2.98%)	6130 (3.01%)	6107 (3.01%)	18694 (8.50%)	7133 (3.45%)	7123 (3.47%)
**Eukaryota**	4849 (2.37%)	4846 (2.38%)	4842 (2.38%)	4852 (2.21%)	4850 (2.35%)	4852 (2.36%)
**Viruses**	231 (0.11%)	231 (0.11%)	232 (0.11%)	364 (0.17%)	234 (0.11%)	234 (0.11%)
**Others**	193715 (94.53%)	192292 (94.48%)	191986 (94.48%)	195945 (89.11%)	194527 (94.08%)	192923 (94.04%)

**Table 4:** Statistics of annotation data.

Databases	Numbers of genes annotated
**Nr**	24,751
**KEGG**	27,445
**eggNOG**	30,443
**CAZy**	1,504
**CARD**	210
**AROs**	190

In summary, these metagenomic data offer compelling evidence on the composition and function of intestinal endosymbionts from *D. citri*, which not only give further comprehension for gut-associated endosymbionts and host insect coevolution under distinguished ecological niches but also open a new avenue to pest management.

## Methods

### Psyllid sampling, tissue collection and DNA isolation

Adult *D. citri* newly emerged (five days old) was initially harvested from *Citrus Tachibana* uninfected *C*Las in 2020 at Guangxi province, China, and was successively reared on *Murraya paniculata* at Guangxi Special Crops Research Institute for more than 15 generations. All cages and experimental treatment were maintained on the conditions of 27 ± 1 °C, 60–70% RH, and at 14:10 h of photoperiod. It was confirmed by PCR detection that *D. citri* did not carry *C*Las.

A field population of *D. citri* newly emerged (five days old) infected *C*Las was collected from Guilin City, China, in 2022. Ninety adult psyllids from *Citrus Tachibana* infected *C*Las were randomly collected for calculating the infection rate. DNA extraction of one adult psyllid followed by Yu *et al*.^12^. with slight modifications. Briefly, genome DNA was extracted using the QIAGEN DNeasy Kit (QIAGEN, Hilden, Germany) according to the manufacturer’s instructions. Subsequently, HLB pathogen-specific primer set OI1/OI2c (forward primer 50-GCGCGTATGCAATACGAGCGGCA-30 and reverse primer 50-GCCTCGCGACTTCGCAACCCAT-30) were performed to amplify the target fragment. Afterward, the *C*Las infection rate was evaluated by agarose gel electrophoresis. When the *C*Las-infected proportion reached over 80%, *D. citri* was considered to be the *C*Las-infected population^12^.

To eliminate the host plant microbial endosymbionts contamination, a 6 h starvation treatment was necessary. For the metagenome sequencing, one hundred and fifty newly emerged (five days old) *C*Las-free and *C*Las-infected *D. citri* respectively were surface sterilized with 70% ethanol for 60 s and rinsed three times with sterile water^13^, with three biological replicates. Then all samples were dissected to obtain the intestinal tissue, which was homogenized with 200 ml sterile water, and frozen at - 80 °C before DNA extraction. The QIAGEN DNeasy Kit was used for microbial genomic DNA extraction. The DNA quality checking and concentrations quantification were performed by Agilent Bioanalyzer 2100 system. The high-quality DNA was sent to Novogene Company (Beijing, China) for metagenomics sequence analyses.

### Metagenome sequence

NEBNext^®^ Ultra^TM^ DNA Library Prep Kit for Illumina (NEB, USA) was used to construct the library. The genomic DNA was randomly sheared into short fragments which were end-repaired, A-tailed, and further ligated with an Illumina adapter. Then fragments with adapters were PCR amplified, size selected, and purified to complete library establishment. The library quality was checked with Qubit 2.0 Fluorometer (Thermo Fisher Scientific, Massachusetts, USA). Library quantification and size distribution detected by real-time PCR and bioanalyzer. Quantified libraries were pooled and sequenced on Illumina PE150 platforms, according to effective library concentration and data amount required.

Readfq (V8, https://github.com/cjfields/readfq) was used for preprocessing raw data from the Illumina sequencing platform to obtain clean data for subsequent analysis^14^. Then MEGAHIT software (v1.0.4-beta) was used for assembly analysis of clean data, and scaftigs without N were obtained by breaking the resulted scaffolds from the N junction^15,16^. MetaGeneMark (V3.05, http://topaz.gatech.edu/GeneMark/) was used to perform ORF prediction for Scaftigs (> = 500 bp) of each sample^14,17,18^. For the ORF prediction results, CD-HIT software (V4.5.8, http://www.bioinformatics.org/cd-hit/) was used to eliminate redundancy and obtain the non-redundant initial gene catalogue^19^. Clean data of each sample were aligned with the initial gene catalog using Bowtie2 (Bowtie2.2.4, http://bowtie-bio.sourceforge.net/bowtie2/index.shtml) to calculate the number of reads of the genes on each sample alignment. The abundance of each gene in each sample was calculated based on the number of reads aligned and the length of gene^20^.

### Metagenome annotation

DIAMOND^21^ (V0.9.9, https://github.com/bbuchfink/diamond/) was used for the alignment of unigenes sequences with those of bacteria, fungi, archaea, and viruses extracted from NCBI’s NR database (Version 2018-01-02, https://www.ncbi.nlm.nih.gov/), and LCA algorithm was adopted to determine the species annotation information of the sequence. Out of the results of the LCA annotation and gene abundance table, the abundance of each sample at each taxonomy and the corresponding gene abundance tables were acquired^14,18,22^. Based on the abundance tables at each taxonomy level, Krona analysis^23^, relative abundance overview, and abundance clustering heatmap were performed, combined with PCA^24^ (R ade4 package, Version 3.2.1) and NMDS^25^ (R vegan package, Version 2.15.3) analysis of dimension reduction. LEfSe analysis was used to search for species differences between groups^26^.

DIAMOND software was used to align unigenes with those in the functional database, including the KEGG database (Version 2018-01-01, http://www.kegg.jp/kegg/)^27,28^, eggNOG database (Version 4.5, http://eggnogdb.embl.de/#/app/home)^29^, and CAZy database (Version 201801, http://www.cazy.org/)^30^. According to the alignment results, the relative abundance at different functional levels was calculated. Based on the abundance table at each taxonomy level, annotated genes statistics, relative abundance overview, and abundance clustering heat map were carried out, combined with PCA and NMDS analysis of dimension reduction, metabolic pathway comparative analysis, as well as Metastatic and LEfSe analysis on inter-group functional differences.

## Data Records

The raw metagenomic sequencing data (fastq format) were deposited in NCBI’s Sequence Read Archive (PRJNA933393)^31–36^.

## Technical Validation

Contaminants and trim reads of the metagenomic sequencing data were removed and the clean reads accounted for more than 99% (Fig. 2), illustrating that the data had incredible quality. To ensure unbiased data production, Randomization principles were carried out and abided by sample collection, extraction, and *C*Las infection rate detection. Using several pieces of software, we confirmed the technical validation of the taxonomic assignments, completeness, and function annotations.

## Data Availability

No custom code was used to generate or process these data.
